# Neuroprotection Against Oxidative Stress: Phytochemicals Targeting TrkB Signaling and the Nrf2-ARE Antioxidant System

**DOI:** 10.3389/fnmol.2020.00116

**Published:** 2020-07-02

**Authors:** Md. Abdul Hannan, Raju Dash, Abdullah Al Mamun Sohag, Md. Nazmul Haque, Il Soo Moon

**Affiliations:** ^1^Department of Anatomy, Dongguk University College of Medicine, Gyeongju, South Korea; ^2^Department of Biochemistry and Molecular Biology, Bangladesh Agricultural University, Mymensingh, Bangladesh; ^3^Department of Fisheries Biology and Genetics, Patuakhali Science and Technology University, Patuakhali, Bangladesh

**Keywords:** neuroprotection, oxidative stress, Nrf2-ARE system, TrkB/PI3K signaling, phytochemicals, neurodegeneration

## Abstract

Oxidative stress (OS) plays a critical role in the pathophysiology of several brain-related disorders, including neurodegenerative diseases and ischemic stroke, which are the major causes of dementia. The Nrf2-ARE (nuclear factor erythroid 2-related factor 2/antioxidant responsive element antioxidant) system, the primary cellular defense against OS, plays an essential role in neuroprotection by regulating the expressions of antioxidant molecules and enzymes. However, simultaneous events resulting in the overproduction of reactive oxygen species (ROS) and deregulation of the Nrf2-ARE system damage essential cell components and cause loss of neuron structural and functional integrity. On the other hand, TrkB (tropomyosin-related kinase B) signaling, a classical neurotrophin signaling pathway, regulates neuronal survival and synaptic plasticity, which play pivotal roles in memory and cognition. Also, TrkB signaling, specifically the TrkB/PI3K/Akt (TrkB/phosphatidylinositol 3 kinase/protein kinase B) pathway promotes the activation and nuclear translocation of Nrf2, and thus, confers neuroprotection against OS. However, the TrkB signaling pathway is also known to be downregulated in brain disorders due to lack of neurotrophin support. Therefore, activations of TrkB and the Nrf2-ARE signaling system offer a potential approach to the design of novel therapeutic agents for brain disorders. Here, we briefly overview the development of OS and the association between OS and the pathogenesis of neurodegenerative diseases and brain injury. We propose the cellular antioxidant defense and TrkB signaling-mediated cell survival systems be considered pharmacological targets for the treatment of neurodegenerative diseases, and review the literature on the neuroprotective effects of phytochemicals that can co-activate these neuronal defense systems.

## Introduction

Oxidative stress (OS) is a pathological condition resulting from an imbalance between ROS generation and cellular antioxidant capacity. Factors contributing to OS in the brain include excitotoxicity, cellular antioxidant system depletion, lipid-rich membranes, susceptibility to lipid peroxidation, and high oxygen demand (Sivandzade et al., 2019). Excess ROS causes structural and functional modifications of cellular biomolecules, including proteins, DNA, and lipids, and thus potentially limits neuronal function and survival. The mechanisms underlying the pathobiologies of neurodegenerative diseases (NDDs) remain elusive; however, evidence strongly suggests a significant relationship between OS and NDDs, such as Alzheimer’s disease (AD) and Parkinson’s disease (PD) (Niedzielska et al., 2016). In addition, OS is known to contribute to the pathogeneses of secondary damage after cerebral ischemia and other brain injuries (Rodrigo et al., 2013; Rodríguez-Rodríguez et al., 2014).

The deposition of misfolded proteins, as is evident in major NDDs, can also induce inflammatory responses, which promote ROS generation and result in OS (Liu et al., 2017). Furthermore, OS causes and is caused by mitochondrial dysfunction (Wang et al., 2014). Given the central role mitochondria play in energy metabolism and the regulation of redox homeostasis, this dysfunction could contribute to the pathobiologies of brain disorders. Howsoever caused, when encountered, cells compensate for the damaging effect of OS by activating the intracellular antioxidant defense system, which is unfortunately compromised in a background of NDD. Therefore, it would appear triggering this endogenous defense system by activating Nrf2 might provide a means of suppressing OS-mediated cellular damage. However, although OS damages neuronal cytoarchitecture, minimizing the detrimental effect of ROS alone may not suffice to prevent/reverse OS-mediated cellular damage, which suggests approaches that help regenerate damaged neuronal structures should also be considered. Physiologically, neuronal growth and survival are maintained via the neurotrophic signaling pathway, but an alteration in the regulation of specific neurotrophic factors and their receptors ensued in the degenerating and aging brains (Sampaio et al., 2017). In particular, the brain-derived neurotrophic factor (BDNF)-dependent TrkB pathway, which is an essential signaling pathway for the survival and normal functioning of mature neurons, is compromised due to lack of BDNF (Gupta et al., 2013; Mitre et al., 2017). These relationships suggest the TrkB pathway and the Nrf2 signaling system are potential targets for promoting neuronal survival and initiating the regeneration of damaged neuronal structures and synaptic connectivity.

Phytochemicals and other natural products can directly scavenge oxygen free radicals and enhance the expressions of cellular antioxidant enzymes and molecules (Amato et al., 2019), and thus, protect against OS-mediated cellular injury (Son et al., 2008; Lee et al., 2014; Naoi et al., 2017; Hannan et al., 2020b). These bioactive compounds and their natural sources have been demonstrated to have neuritogenic potentials (Jang et al., 2010; Hannan et al., 2013, 2014, 2019) and to aid the reconstruction of synaptic connectivity by regenerating damaged neuronal processes (Moosavi et al., 2015; Venkatesan et al., 2015; Tohda, 2016). In fact, several studies have described a number of natural pharmacological modulators that can co-activate antioxidant defense and neurotrophin signaling-mediated cell survival systems (Gao et al., 2015; Kwon et al., 2015; Zhang et al., 2017; Cui et al., 2018; Fang et al., 2018; Hui et al., 2018), and suggested that these compounds have therapeutic potential for the treatment of OS-mediated brain disorders. Targeting both of these signaling systems with a single compound offers some benefits over that with combination. For example, the first strategy could bypass the possible drug–drug interactions that could be either synergistic or antagonistic. Instead, if a single compound can activate both the signaling system, it would be more convenient to establish it as a therapeutic agent concerning pharmacokinetics and drug delivery. However, we did not ignore the dual targeting with a combination that has also some other pharmacological benefits. While there is a sizable quantity of natural products that independently activate either TrkB signaling or Nrf2 pathway (Moosavi et al., 2015; Murphy and Park, 2017), we limited our review only to those reports that describe co-activation of TrkB signaling and Nrf2-ARE antioxidant pathways.

In this review, we provide a brief overview of the causes of OS development and its involvement in the pathobiology of NDD and brain injury. We then present the cellular antioxidant defense system and TrkB signaling pathway as pharmacological targets for the treatment of NDDs. Finally, we review recent literature on the neuroprotective effects and underlying pharmacological mechanisms of bioactive phytochemicals that co-activate these neuronal defense systems.

## Oxidative Stress

The metabolisms of all eukaryotes essentially require oxygen to maintain their physiological functions and meet energy demands, but this life-sustaining element can sometimes damage cells, particularly high oxygen consuming cells. Different tissues have different oxygen demands, which are largely determined by metabolic needs. In mammals, the brain, which is a metabolically demanding organ, accounts for around 20% of total oxygen utilization (Halliwell, 2006), and neurons and astrocytes are principally responsible for this oxygen consumption. This huge oxygen turnover often results in the generation of excess ROS [superoxide (O_2⋅–_), the hydroxyl radical (⋅OH), and hydrogen peroxide (H_2_O_2_)]. Moreover, high susceptibility to lipid peroxidation and relatively weaker antioxidant defense leave the lipid-rich brain vulnerable to OS. The unpaired valence electrons of ROS indicate they are highly reactive and capable of damaging to cellular biomolecules (Kim et al., 2015). Although O_2⋅–_ has been suggested to play a central role in the production of ROS, ⋅OH is mainly responsible for its cytotoxic effects (Bolisetty and Jaimes, 2013).

ROS in brain may be of exogenous origin, for example, produced by xenobiotic metabolism or radiation, or of endogenous origin resulting from the activities of ROS-generating enzymes (Kim et al., 2015). The primary sources of ROS are mitochondrial oxidative phosphorylation, particularly by complex I and several redox enzymes (Zorov et al., 2014). Physiologically, a certain amount of ROS is essential for cell signaling and pro-survival pathways (Patten et al., 2010), but when ROS production overwhelms the cellular antioxidant defense system, cells are exposed to the pathological condition termed OS (Gao et al., 2018), which may lead to mitochondrial dysfunction and further ROS generation (Stelmashook et al., 2019). The endoplasmic reticulum, the primary site of protein folding, also involved in ROS generation (Chaudhari et al., 2014), and resulting protein misfolding causes ER stress and additional ROS overproduction (Lindholm et al., 2006).

**FIGURE 1:**
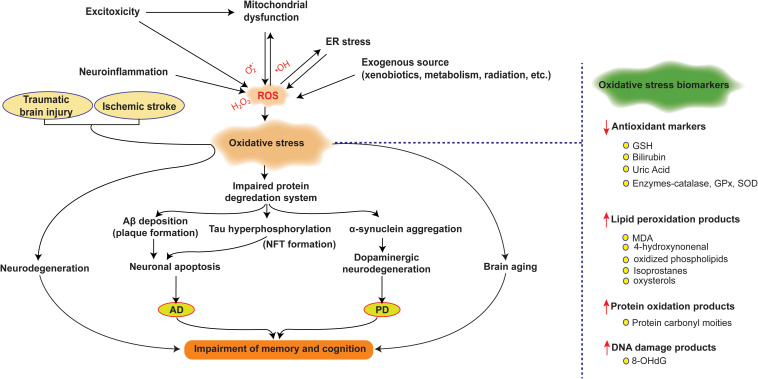
Oxidative stress and its implications in the pathobiologies of neurodegeneration in NDDs and after ischemia or TBI. ROS are produced by multiple sources: endogenously, result from excitotoxic insults, neuroinflammation, ER stress, or mitochondrial dysfunction, whereas exogenously ROS are generated by radiation or xenobiotics. When the production of ROS overwhelms intracellular antioxidant defense, brain cells are exposed to oxidative stress (OS), which may lead to mitochondrial dysfunction and further ROS production. OS impairs the protein degradation system, and thus, hinders the clearance and results in the subsequent deposition of misfolded protein, which in turn, result in lipid peroxidation, protein oxidation, and DNA damage, leading to neuronal death. These events constitute the pathological basis of neurodegenerative diseases (NDDs) and brain aging. OS also contributes to the pathogeneses of secondary damage after cerebral ischemia or other brain injuries. Oxidative metabolites such as the lipid peroxidation products (4-HNE and MDA), protein oxidation products (protein carbonyl moieties), DNA oxidation product (8-OHdG) and antioxidant components such as GSH, bilirubin, uric acid, and antioxidative enzymes (CAT, GPx and SOD) are potential oxidative biomarkers and are elevated in patients with NDDs. OS, oxidative stress; ER, endoplasmic reticulum; NFT, neurofibrillary tangle; AD, Alzheimer’s disease; PD, Parkinson’s disease; GSH, glutathione; GPx, glutathione peroxidase; SOD; superoxide dismutase; CAT, catalase; MDA, malondialdehyde; 8-OHdG, 8-hydroxy-2′-deoxyguanosine, NDD, neurodegenerative disease.

ROS damages cells by compromising the structures and functions of biomolecules, such as by peroxidizing lipids, oxidizing proteins, and damaging DNA (Kim et al., 2015), and eventually causing neurodegeneration (Gandhi and Abramov, 2012). Oxidative metabolites such as lipid peroxidation products [4-hydroxynonenal, 4-HNE, and malondialdehyde (MDA)], protein oxidation products (protein carbonyl moieties), and the DNA oxidation product (8-hydroxy-2′-deoxyguanosine, 8-OHdG) are the biomarkers that are elevated in the patients with NDDs (Zou et al., 2008; Rani et al., 2017; Haque et al., 2019) (Figure 1).

## Oxidative Stress in Cases of Neurodegenerative Diseases or Brain Injury

Dementia disorders, including NDDs, and complications arising from ischemic stroke or traumatic brain injury (TBI), are major public health concerns that are intimately associated with OS. Preclinical and clinical studies have revealed that brain and peripheral tissues and body fluids from patients with these brain disorders contain significantly higher concentrations of OS biomarkers and lower amounts of antioxidant biomarkers (Gandhi and Abramov, 2012).

### Alzheimer’s Disease

Alzheimer’s disease is the most common progressive NDD and the major cause of dementia (Butterfield and Halliwell, 2019). The main pathological hallmarks of AD, include extracellular deposition of amyloid plaque, intraneuronal aggregation of neurofibrillary tangles (NFTs), and brain atrophy (van der Kant et al., 2020). Furthermore, OS has been shown to provoke Aβ deposition (plaque formation), tau hyperphosphorylation (NFT formation), and the subsequent degenerations of synaptic connectivity and neurons by impairing the protein degradation system (Butterfield and Halliwell, 2019). Several studies have reported elevated levels of ROS-mediated changes in AD brains, which supports the notion that OS is implicated in the pathobiology of AD (Wojsiat et al., 2018; Youssef et al., 2018). For example, levels of MDA and 4-HNE (measures of lipid peroxidation) are higher than normal in the brain tissues and cerebrospinal fluid samples of AD patients (Di Domenico et al., 2017; Butterfield and Boyd-Kimball, 2018). Although 4-HNE levels remained unchanged, the levels of antioxidant enzymes, such as superoxide dismutase (SOD), glutathione peroxidase (GPx), catalase (CAT), and peroxiredoxin (Prdx) were altered in the affected areas of the brain (Youssef et al., 2018). High plasma levels of protein carbonyls and advanced glycation end products (carboxymethyllysine and carboxyethyllysine) have been detected in male AD patients (Sharma et al., 2020). Moreover, 3-nitrotyrosine (3-NT), a protein nitration product, was found to be increased in CD3^+^ T-cells from AD patients (Tramutola et al., 2018). In AD patients, reductions in the activities of antioxidant enzymes result in a significant decline in plasma levels of antioxidants (e.g., uric acid and bilirubin) (Kim et al., 2006). Redox proteomics studies have also reported oxidation of protein moieties in AD models (Di Domenico et al., 2016; Butterfield and Boyd-Kimball, 2019).

Oxidative stress leads to mitochondrial dysfunction and cellular atrophy (Singh et al., 2019) as pathological aggregations of proteins such as Aβ and tau have been reported to target mitochondria and augment ROS production (Buendia et al., 2016). OS also retards synaptic plasticity, and thus, contributes to progressive memory impairment, which is a characteristic clinical symptom of AD (Tönnies and Trushina, 2017). This relationship between OS and AD strongly suggests that strategies linked to antioxidant or antioxidant defense system could play important roles in the future management of AD.

### Parkinson’s Disease

In terms of its prevalence, PD is second only to AD and is characterized by dopaminergic neuron degeneration in the substantia nigra (Gandhi and Abramov, 2012). The intraneuronal aggregation of α-synuclein and the formation of Lewy bodies is a major pathological hallmark of PD (Singh et al., 2019). Although the exact mechanisms underlying the pathophysiology of this disease remains elusive, convincing evidence suggests the crucial involvement of OS (Gaki and Papavassiliou, 2014). Numerous studies have reported elevated levels of oxidative damage markers and low levels of glutathione (GSH) in the substantia nigra of PD patients (Singh et al., 2019). Furthermore, MDA plasma levels (de Farias et al., 2016) and protein carbonyl and 8-OHdG (markers of oxidative damage to protein and DNA, respectively) in brain tissues (Beal, 2002) have been reported to be elevated. A meta-analysis reported elevated levels of 8-OHdG and MDA and reduced levels of catalase, uric acid, and GSH in the blood of PD patients (Wei et al., 2018). These findings support the involvement of OS in the pathobiology of PD and suggest that targeting OS offers a potential therapeutic strategy for addressing this devastating brain disorder.

### Ischemic Stroke

Stroke is the second leading cause of death (Donkor, 2018) and an important cause of permanent disability in adults worldwide (Feigin et al., 2017), and is caused by a sudden interruption in brain blood supply due to vascular occlusion. As a consequence, a portion of the brain experiences oxygen and nutrient insufficiencies, which cause depolarization of neuronal membranes and glutamate surge into synapses, resulting in a cascade of events, including calcium overload, dissipation of mitochondrial membrane potentials, OS, and inflammation (Sivandzade et al., 2019; Hannan et al., 2020a). Inappropriate levels of antiapoptotic proteins [e.g., Bcl-2 (B-cell lymphoma 2)] and proapoptotic proteins [e.g., Bax (Bcl-2-associated X protein)] contribute to mitochondrial dysfunction and OS-induced apoptosis (Wu et al., 2019). In addition, the re-establishment of blood supply immediately after ischemia exposes brain tissue to excess oxygen, which exacerbates ROS production, which, in turn, induces further OS-associated injury, lipid peroxidation, protein oxidation, and intracellular DNA damage (Kishimoto et al., 2019; Sivandzade et al., 2019). Several *in vivo* investigations of OS biomarkers in patients after ischemic stroke suggest that oxidative damage follows the ischemic shock, as blood levels of NO and MDA have been reported to be elevated after ischemic stroke (Dogan et al., 2018). These findings indicate targeting OS offers a promising therapeutic strategy to reduce secondary brain injury after ischemic stroke and to improve outcomes (Sivandzade et al., 2019).

### Traumatic Brain Injury

Traumatic brain injury (TBI) is also a major cause of death and disability worldwide, particularly in countries with high traffic densities. Non-fatal TBI may lead to neurological deficits due to direct tissue damage (primary injury) or subsequent biochemical changes (secondary injury) (Lozano et al., 2015). Primary injuries cause immediate neuronal death, whereas secondary damage leads to progressive neuronal degeneration driven by biochemical factors such as excitotoxicity, inflammation, mitochondrial dysfunction, and OS (Khatri et al., 2018). Thus, it is important that these secondary changes be targeted to reduce further damage. Following TBI, OS markers such as oxidized protein moieties, lipid peroxidation products, and products of DNA damage accumulate in the brain, whereas levels of antioxidant molecules and enzymes, such as GSH, GPx, glutathione reductase (GR), glutathione *S*-transferase (GST), SOD, and CAT decline, which indicates TBI results in OS (Rodríguez-Rodríguez et al., 2014). Neuroprotective strategies directed at salvaging injured brain tissue soon after injury and that promote regeneration during the recovery stage are advantageous (Wurzelmann et al., 2017). The therapeutic potentials of BDNF and its mimics have been reported in the contexts of several neurological conditions, including TBI (Wurzelmann et al., 2017; Houlton et al., 2019). Therefore, it appears targeting cellular antioxidant defense and the BDNF/TrkB signaling pathway might improve cognitive deficits secondary to TBI.

## Cellular Defense Against Oxidative Stress: the Nrf2-Are Antioxidant System

**FIGURE 2:**
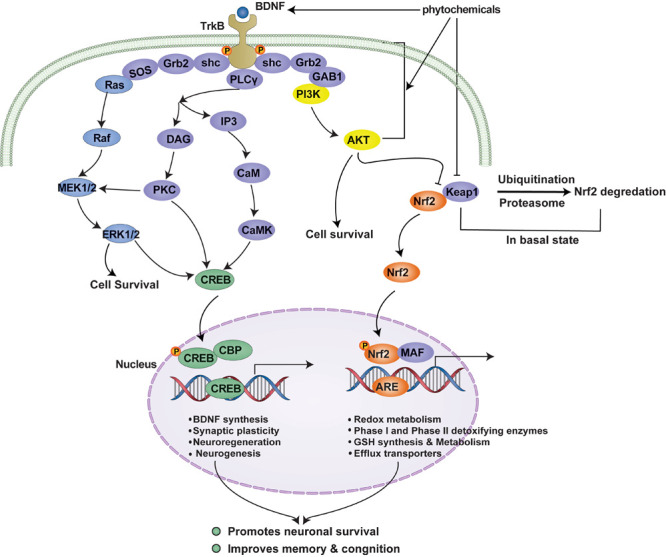
TrkB signaling-mediated cell survival and the Nrf2-ARE antioxidant system. Neurons maintain survival and connectivity through neurotrophin signaling pathway such as the PI3K/Akt, MAPK (Ras/ref/Erk), and PLCγ (PKC or CaMK) pathways. The PI3K/Akt pathway destabilizes Nrf2-Keap1 complex, which under basal conditions leads to the ubiquitination and degradation of Nrf2 by proteasomal system, and thus, promotes the nuclear translocation of Nrf2. This, in turn, activates the Nrf2-ARE antioxidant system and results in the expressions of multiple genes that encode antioxidant enzymes responsible for redox metabolism and GSH synthesis and metabolism. Activated Akt also regulates cell survival by maintaining a balance between pro-apoptotic and anti-apoptotic proteins. Furthermore, MAPK and PLCγ signaling pathways regulate neuronal survival and the transcriptions of CREB-dependent genes that encode BDNF and other proteins required for synaptic plasticity and neurogenesis. Phytochemicals may promote cell survival by activating TrkB signaling by functioning as BDNF mimetics or by promoting Akt phosphorylation or inhibiting Nrf2-Keap1 complex, and thus, activating the antioxidant defense system. TrkB signaling and the Nrf2-ARE antioxidant system are complementary to each other and simultaneous activation of these pathways has been shown to confer neuroprotection against OS and to attenuate memory and cognition impairments in patients with NDDs or brain injury. BDNF, brain-derived neurotrophic factor; PI3K, phosphatidyl inositol-3 kinase; Akt, protein kinase B; MEK1/2, mitogen-activated protein kinase kinase; ERK1/2, extracellular signal-regulated kinase 1/2; PLCγ, phospholipase C-γ; DAG, diacylglycerol; PKC, protein kinase C; IP3, inositol 1,4,5-trisphosphate; CaM, calmodulin; CaMK, Ca^2+^/calmodulin-dependent protein kinase; CREB, cAMP response element-binding protein); CBP, CREB binding protein; CRE, cAMP response elements; Nrf2, nuclear factor erythroid 2-related factor 2; Keap1, Kelch-like ECH-associated protein 1; MAF, small musculoaponeurotic fibrosarcoma; ARE, antioxidant response elements.

Cells are equipped with an antioxidant defense system comprised of antioxidant enzymes and other molecules that attenuate OS-mediated injury. The Keap1-Nrf2 pathway is the principal cellular pathway that regulates the antioxidant defense system. Nrf2 (encoded by *NFE2L2*) is a master regulator of cellular redox homeostasis, and its activity is regulated in various ways. Under resting conditions, Nrf2 is sequestered by Keap1 (Kelch-like ECH-associated protein) in the cytoplasm (Itoh et al., 1999), where it is polyubiquitinated and targeted for proteasomal degradation (McMahon et al., 2003). However, under OS or in a background of pharmacological intervention, the Nrf2-Keap1 complex is disrupted, which leads to the release of Nrf2 and its nuclear translocation (Joshi and Johnson, 2012). Once in the nucleus, Nrf2 forms heterodimers with small musculoaponeurotic fibrosarcoma (MAF) proteins, and these Nrf2-MAF heterodimers recognize an enhancer sequence termed ARE present in the regulatory regions of over 250 genes (Ma, 2013) (Figure 2).

Activation of the Nrf2-ARE pathway via Keap1-Nrf2 disruption results in the expressional up-regulation of multiple genes encoding a network of cooperating enzymes that constitute an antioxidant defense system (Joshi and Johnson, 2012; Dinkova-Kostova et al., 2018). The roles of this antioxidant system include redox homeostasis, involving SOD, CAT, sulfaredoxin (Srx), thioredoxin (Trx), and Prdx; GSH synthesis and metabolism, involving Gpx, GR, γ-glutamine cysteine ligase (GCL) and synthase (GCS); quinone recycling, involving NAD(P)H quinone oxidoreductase (Nqo1), and iron homeostasis- heme oxygenase 1 (HO-1).

Superoxide dismutases mediate the conversion of O_2_⋅^–^ to H_2_O_2_, which is subsequently neutralized to H_2_O. SOD2 (Mn-SOD) is primarily found in mitochondria, where it acts to minimize oxidative damage by O_2_⋅^–^ (Murphy, 2009). Knockout of SOD2 increased amyloid plaque burden and exacerbated cognitive deficits in mice (Li et al., 2004; Esposito et al., 2006; Lee et al., 2012), and conversely, SOD2 overexpression reduced oxidative markers, amyloid deposition, and cognitive deficit in transgenic AD mice (Dumont et al., 2009). Peroxisomal catalase and GPx both catalyze the conversion of H_2_O_2_ to water and oxygen. GSH is generated from glutamate, cysteine, and glycine by γ-GCS, GCL, and glutathione synthetase (GS) (Murphy and Park, 2017). Furthermore, HO-1, in combination with cytochrome p450 and NADPH, catalyzes the degradation of heme to biliverdin, which is subsequently converted to bilirubin by biliverdin reductase, and both biliverdin and biliverdin reductase have antioxidant and anti-inflammatory effects (Joshi and Johnson, 2012; Murphy and Park, 2017). HO-1 overexpression has been reported in NDD (Schipper, 2004), presumably to compensate for oxidative damage (Cuadrado and Rojo, 2008).

In the degenerative brain, Nrf2 is primarily localized in cytoplasm (Ramsey et al., 2007), which suggests Nrf2 activity is insufficient to mediate a competent antioxidant response. This shortfall in Nrf2 activity might explain why oxidative damage is commonly observed in NDDs despite the presence of an antioxidant defense system (Ramsey et al., 2007). Evidence shows that Nrf2 knockdown left neurons more susceptible to OS, whereas Nrf2 overexpression reversed the effect (Lee and Johnson, 2004). In an amyloid protein precursor/presenilin 1 (APP/PS1) mouse model, Nrf2 knockout aggravated oxidative damage (Joshi et al., 2015), whereas activation or overexpression of Nrf2 protected APP/PS1 mice from Aβ toxicity (Kanninen et al., 2008). Moreover, in rat stroke model, Nrf2 overexpression rescued neurons from ischemic shock (Shih et al., 2005). Pharmacological modulators of Nrf2 have also shown therapeutic promise in experimental models of NDD (Dinkova-Kostova et al., 2018). Therefore, it is believed activation of the antioxidant defense system based on the targeting of Nrf2 signaling offers potential means of treating NDDs and brain injuries.

## Cell Survival System: the TrkB Signaling Pathway

Neurotrophin signaling plays an essential role in maintaining neuronal survival and synaptic plasticity as well as learning and memory (Bothwell, 2019). BDNF is predominant among the several neurotrophins in the adult central nervous system. BDNF binds TrkB and helps maintain neuronal survival and synaptic plasticity by activating canonical signaling pathways, that is, the PI3K/Akt pathway, the mitogen-activated protein kinase (MAPK) pathway, and the phospholipase C-γ (PLCγ) pathway (Kowiański et al., 2018). The PI3K/Akt signaling pathway is the major TrkB-mediated survival pathway that promotes neuronal survival and protects against apoptosis (Wu et al., 2019). Activated Akt also controls cell survival by maintaining a balance between pro-apoptotic and anti-apoptotic proteins (Sussman, 2009). The MAPK and PLCγ pathways regulate neuronal growth and survival by expressing multiple genes in a CREB (cAMP response element binding protein)-dependent pathway, which encodes BDNF and other proteins associated with synaptic plasticity (Cunha et al., 2010; Numakawa et al., 2018) (Figure 2).

BDNF heterozygous knockout mice (BDNF ± mice) exhibit fear learning deficits (Meis et al., 2017). Moreover, in AD, alterations in BDNF level and its receptor were observed in the frontal cortex and the entorhinal cortex, which control spatial memory and higher cognitive functions (Weissmiller and Wu, 2012). Also, in *in situ* hybridization of the patient sample, the protein and mRNA levels of BDNF were found to be decreased in dopaminergic neurons of the substantia nigra (Mogi et al., 1999), in which the neurons are most vulnerable in PD. A line of evidence from experimental studies and metanalysis further showed a close correlation between decreased BDNF levels and neuronal loss in neurological disorders (Ventriglia et al., 2013; Siuda et al., 2017; Jiang et al., 2019). These observations suggest that the BDNF/TrkB signaling pathway plays an essential role in neuronal growth, survival and synaptic plasticity. Several natural compounds have been reported to activate the TrkB signaling pathway and promote cell survival (Obianyo and Ye, 2013), and therefore, have been suggested as possible treatments for NDD and brain injury.

## Cross-Talk Between TrkB Signaling Pathway and Nrf2-Are Antioxidant System

Neurons maintain survival and connectivity through neurotrophin signaling pathways such as the PI3K/Akt, MAPK (Ras/ref/Erk), and PLCγ (PKC or CaMK) pathways. A piece of evidence suggests that the TrkB signaling pathway, the dominant neurotrophin pathway in mature neurons, may act upstream of the Nrf2-ARE system. For example, phosphorylation of Akt mediates the nuclear translocation of Nrf2, which, in turn, activates the Nrf2-ARE system resulting in the transcriptions of multiple genes encoding antioxidant enzymes (Qi et al., 2017). Moreover, PI3K/Akt pathway regulates hemeoxygenase-1 (HO-1), which is involved in the maintenance of cellular homeostasis (Pischke et al., 2005). Besides, growing evidence suggests a possible cross-talk between the TrkB signaling pathway and the Nrf2-ARE antioxidant system. For instance, the MAPK (ERK/p38 MAPK) pathway, another downstream signaling of TrkB pathway, was shown to regulate Nrf2 transcriptional activity (Singh et al., 2010; Tufekci et al., 2011). TrkB signaling pathway and the Nrf2-ARE antioxidant system are, therefore, complementary to each other and simultaneous activation of these pathways has been shown to confer neuroprotection against OS and to attenuate memory and cognition impairments in patients with NDDs or brain injury.

Brain-derived neurotrophic factor-dependent p75^NTR^ signaling is associated with TrkB and may also activate Nrf2 pathway. The primary receptor of BDNF, TrkB, has two functional isoforms-TrkB.FL (full-length) and TrkB.T1 (truncated) receptors. TrkB.FL inhibits ceramide generation through its low-affinity receptor, p75^NTR^, via its tyrosine kinase activity. Conversely, TrkB.T1, lacking the intracellular tyrosine kinase domain, promotes ceramide generation following activation by BDNF. The p75^NTR^ signaling pathway may act as a double-edged sword (Ishii and Mann, 2018). The overstimulation of p75^NTR^ results in excess ceramide generation and was known to be associated with apoptosis (Ibáñez and Simi, 2012; Shen et al., 2019). Whereas, TrkB.FL receptor tyrosine kinase activity inhibits sphingomyelinase protecting cells from ceramide toxicity along with restricting the p75^NTR^-mediated prodeath signaling pathway (Ishii et al., 2018). Moreover, the BDNF-TrkB.T1-p75^NTR^ signaling complex generates physiological concentrations of ceramide to activate protein kinase Cζ (PKCζ) leading to activation of casein kinase 2 (CK2) and Nrf2, thereby modulating antioxidant capacity of cells (Ishii et al., 2018).

Neurons express both TrkB.FL and TrkB.T1 receptors but the ratio of these receptor levels changes based on the neuronal activity (Gomes et al., 2012). The excitotoxic stimulation of cultured rat hippocampal neurons results in the downregulation of TrkB.FL, while upregulation of TrkB.T1 expression caused a significant alternation in the ratio of the two receptors (Gomes et al., 2012), which allows BDNF to induce Nrf2 activation in the presence of p75^NTR^ (Ishii and Mann, 2018). This mechanism protects neurons from oxidative damage during excitotoxic stimulation with glutamate, an event that frequently encountered in neural injury, stroke, and NDDs (Ishii and Mann, 2018). Moreover, BDNF-TrkB.T1-p75^NTR^-Nrf2-mediated neuroprotection is context-dependent (Bothwell, 2019), i.e., it depends on the degree of activation. However, there is no evidence, so far, demonstrating the association of the neuroprotective phytochemicals with this signaling pathway suggesting further investigation.

## Phytochemicals That Activate Neuronal Antioxidant Defense and Survival

Many plant-derived bioactive molecules neutralize ROS and reportedly, potentiate the cellular antioxidant system. This latter mode of inducing an antioxidant effect by promoting adaptive cellular stress response using phytochemicals is substantially supported (Son et al., 2008; Lee et al., 2014). Furthermore, these molecules can promote cellular survival by activating the growth signaling pathway. Here, we reviewed the literature over the past 5 years for phytochemicals that have been shown to protect neurons from OS by activating TrkB signaling pathways and the Nrf2-ARE system; two excellent reviews appropriately addressed preceding reports (Moosavi et al., 2015; Murphy and Park, 2017). Specific targets, experimental and disease models, research outcomes, and pharmacological markers of these phytochemicals are summarized in Tables 1–3.

**TABLE 1:** Neuroprotection afforded by phytochemicals targeting the PI3K/Akt/Nrf2 signaling pathway against AD and other neurodegenerative disorders.

Modulator	Specific target pathway	Chemical class and natural sources	Experimental model	Disease model	Pathobiology involved	Major research outcomes	Molecular markers	References
**Phenolic compounds**								
Sulfuretin	PI3K/Akt and Nrf2/HO-1 signaling pathways	Flavonoid glycosides; stem bark of *Albizzia julibrissin* and heartwood of *Rhus verniciflua*	Aβ-induced neurotoxicity in SH-SY5Y cells and primary hippocampal neurons	AD	Oxidative stress	Neuroprotection (antioxidation and increased cell survival)	↓ROS, ↑HO-1 ↑PI3K/Akt ↑Nrf2	Kwon et al. (2015)
Resveratrol	PI3K/Akt/Nrf2 pathway	Polyphenol; grapes	Aβ1–42-induced cytotoxicity in PC12 cells	AD	Oxidative stress	Neuroprotection (antioxidant and anti-apoptosis); amelioration of memory impairment	↓MDA, ROS ↑SOD, HO-1, GSH, Nrf2 ↑PI3K, Akt	Hui et al. (2018)
Anthocyanins	PI3K/Akt/Nrf2 signaling	Anthocyanins; Korean black beans	APP/PS1 mouse model of AD; AβO-induced neurotoxicity in HT22 cells	AD	Oxidative stress	Neuroprotection (antioxidant)	↑p-PI3K, p-Akt, pGSK3β (Ser9) ↑Nrf2 ↑HO-1, GCLM ↓MDA, H_2_O_2_, 8-OxoG ↑GSH ↓cleaved caspase 3 ↓PARP1	Ali et al. (2018)
Tea polyphenols	TrkB/CREB/BDNF pathway and Keap1/Nrf2 signaling pathway	Tea flavonoids	H_2_O_2_-treated SH-SY5Y neuronal cells; shift work disruption model of C57BL/6J male mice	NDD	Oxidative stress	Neuroprotection (antioxidation, anti-apoptosis, attenuates mitochondrial dysfunction); amelioration of memory impairment	↓Bax, cytochrome c, caspase-3 activation, PARP cleavage, ↑Bcl-2 ↑p-TrkB, pCREB, BDNF ↑MMP, ↓H_2_O_2_ ↓IκB, NF-κB ↑ERK, pAkt, pERK ↓pJNK, pP38 ↑HO-1, NQO-1 c, Nrf2, Keap1 ↑γGCS, MnSOD, GPx1, GSH, SOD, CAT	Qi et al. (2017)
8-Hydroxydaidzein	Nrf2-antioxidant and Akt/NF-κB-inflammatory signaling pathways	Isoflavone; fermented soy food	LPS-stimulated BV2 microglial cells	NDD	Neuroinflammation and oxidative stress	Anti-inflammation and antioxidation	↓NO ↓TNF-α, IL-6, IL-1β↓ROS, ↑Nrf2 ↑HO-1, NQO1 ↓NF-κB-p65 ↓PGE2	Wu et al. (2018)
Rutin	PI3K/Akt/GSK-3β/NRF-2 signaling pathway	Flavonoid; buckwheat	Acrylamide or γ-radiation-induced neurotoxicity in male albino SD rats	NDD	Oxidative stress	Neuroprotection (antioxidant and anti-inflammatory)	↑p-PI3K, p-Akt, p-GSK-3β↑NRF-2, ↓MDA ↓GST ↓IL-1b, IL-6 ↑IGF1, NGF	Thabet and Moustafa (2018)
**Non-phenolic compounds**								
Brassicaphenanthrene A	Nrf2-mediated HO-1 expression by PI3K/Akt and JNK regulatory pathways	Phenanthrene derivative; *Brassica rapa* ssp. campestris (Brassicaceae) (Turnip)	Glutamate-induced excitotoxicity in HT-22 neuronal cells	AD	Oxidative stress	Neuroprotection (antioxidation)	↑HO-1, Nrf2 ↑GSH ↑Glutamine- cysteine ligase ↑Nrf2 nuclear translocation and ARE promoter activity ↑pAkt	Lee et al. (2020)
Acerogenin A	PI3K/Akt/Nrf2/HO-1 pathway	Stem bark of Japanese folk medicine *Acer nikoense*	Glutamate-induced oxidative neurotoxicity in HT22 cell line	NDD	Oxidative stress	Neuroprotection (antioxidant)	↓ROS ↑HO-1 ↑Nrf2 ↑pAkt	Lee et al. (2015)
TMC-256C1	PI3K/Akt/Nrf2 pathway	Marine-derived fungus *Aspergillus* sp. SF6354	LPS-stimulated BV2 microglial cells and glutamate-induced neurotoxicity in mouse hippocampal HT22 cells	NDD	Oxidative stress and neuroinflammation	Neuroprotection (anti-inflammatory and antioxidant)	↑HO-1, Nrf2 ↓ROS ↓TNF-α, IL-1β, IL-6, IL-12 ↓I PEG_2_, NO ↓Cox2, iNOS ↓NF-κB, pIκBα, p65, p50 ↑pAkt	Kim et al. (2016)
Polysaccharide extracts	PI3K/Akt and Nrf2-mediated HO-1/NQO1 pathways	Polysaccharide; *Perilla frutescens*	H_2_O_2_-induced oxidative stress in HT22 hippocampus cells	NDD	Oxidative stress	Neuroprotection (antioxidant and anti-inflammatory)	↓Bax, cytochrome c ↓caspases-3, -8, and -9 ↑PARP, ↑Bcl-2 ↑SOD, ↓MDA ↓p MAPKs (p38, ERK, JNK), ↓NF-κB ↑HO-1, ↑p-PI3K, ↑pAkt, p65	Byun et al. (2018)
3,3’-Diindolylmethane	TrkB/Akt pathway and antioxidant enzyme system	Metabolite of indole-3-carbinol; Brassicaceae vegetables	Glutamate-treated HT-22 cells; scopolamine-treated ICR mice	NDD	Oxidative stress	Neuroprotection (antioxidant and anti-apoptosis); amelioration of memory impairment	↓ROS, ↑GSH ↓Bax, cytochrome c, cleaved caspase-3, AIF ↑Bcl-2 ↑p-TrkB, p-CREB, BDNF, p-Akt ↑HO-1, GCLC, NQO-1 ↓MDA ↓AChE, ↑ChAT ↑GR, Gpx	Lee et al. (2019)
*N*-Acetyl serotonin	BDNF/TrkB/CREB signaling and Akt/Nrf2/antioxidant enzyme pathway	Naturally occurring precursor and intermediate in the melatonin biosynthesis	Glutamate or H_2_O_2_-induced oxidative stress in HT-22 cells; scopolamine-treated memory impairment in Swiss CD-1 mice	AD	Oxidative stress	Neuroprotection (antioxidation and anti-apoptosis); amelioration of memory impairment	↓AIF, Bax, calpain, cytochrome c and cleaved caspase-3 ↑Bcl-2 ↑pTrkB, pCREB, BDNF ↓ROS, ↑GSH ↑Nrf2, HO-1, GCLC	Yoo et al. (2017)

**TABLE 2:** Neuroprotection afforded by phytochemicals targeting the PI3K/Akt/Nrf2 signaling pathway against ischemic stroke and other neuronal injuries.

Modulator	Specific target pathway	Chemical class and natural sources	Experimental model	Disease model	Pathobiology involved	Major research outcomes	Molecular markers	References
**Phenolic compounds**								
Totarol	Akt/HO-1 pathway	Phenolic diterpenoid; sap of *Podocarpus totara*	Glutamate and OGD-induced injury in rat cerebellar granule neurons and cerebral cortical neurons; MCAO model of acute cerebral ischemic injury in adult male SD rats	Ischemic stroke	Oxidative stress	Neuroprotection (antioxidant)	↑pAkt, pGSK-3β↑Nrf2 ↑HO-1, SOD ↑GSH	Gao et al. (2015)
Rosmarinic acid	PI3K/Akt/Nrf2 signaling pathway	Phenolic compound; commercial source	Right middle cerebral artery occlusion in CD-1 mice	Ischemic stroke	Oxidative stress	Neuroprotection (anti-oxidative and anti-apoptotic properties)	↑Bcl-2, ↓Bax ↑HO-1, ↑Nrf2 ↑SOD, ↓MDA ↓pAkt	Cui et al. (2018)
Baicalin	Akt/Nrf2 pathway	Flavone; radix of *Scutellaria baicalensis*	TBI mice model	TBI	Oxidative stress	Neuroprotection (anti-oxidative and anti-apoptotic properties); attenuates neurological deficits and brain edema	↑Bcl-2, ↓Bax, ↓MDA ↑GPx, SOD, NQO-1, HO-1 ↓cleaved caspase 3 ↑pAkt	Fang et al. (2018)
**Non-phenolic compounds**								
Diallyl trisulfide	PI3K/Akt -mediated Nrf2/HO-1 signaling pathway	Organosulfur compound of garlic oil	OGD-induced neuronal injury in B35 rat neuroblastoma cells	Ischemic stroke	Oxidative stress	Neuroprotection (antioxidant)	↓ROS ↓MDA ↑Nrf2 ↑HO-1 ↓Cleaved caspase-3 ↑pAkt	Xu et al. (2015)
Oxymatrine	Akt/GSK3β/HO-1/Nrf-2 signaling pathway	Quinolizidine alkaloid; Chinese herb *Sophora flavescens*	Hypoxic-ischemic brain injury model of P7 SD rats	Ischemic stroke	Oxidative stress	Neuroprotection (anti-oxidative and anti-apoptotic properties); attenuates neurological deficits and reduces infarct volume	↑p-Akt ↑p-GSK3β↑Nrf-2 ↑HO1	Ge et al. (2018)
6′-*O*-galloylpaeoniflorin	PI3K/Akt/Nrf2 activation	Galloylated derivative of paeoniflorin; peony root	OGD-induced ischemic model of PC12 cells; CIRI model of male Wistar rats	Ischemic stroke	Neuroinflammation and oxidative stress	Neuroprotection (antioxidant and anti-inflammatory); reduces infarct volume and improves neurological deficits	↑p-Akt, Nrf2 ↓MDA, SOD ↓TNF-α, IL-1β↓Caspase 3	Wen et al. (2018)
Diterpene ginkgolides (ginkgolides A, B and C)	Akt/Nrf2 and Akt/CREB signaling pathways	Ginkgolide terpenoid lactones; *Ginkgo biloba* L	MCAO model of acute cerebral ischemic injury in adult male SD rats; OGD/R-induced ischemic injury in PC12 cells	Ischemic stroke	Oxidative stress	Neuroprotection (antioxidant)	↑pAkt, pNrf2, pCREB ↑HO-1 ↓cleaved caspase 3, Bax	Zhang et al. (2018)
Ginkgolides (ginkgolide A, ginkgolide B, ginkgolide K) and bilobalide	Akt/Nrf2 signaling pathway	Ginkgolide terpenoid lactones; *Ginkgo biloba* L	OGD-induced ischemic model of SH-SY5Y cells; MCAO model of cerebral ischemic injury in male SD rats	Ischemic stroke	Oxidative stress	Neuroprotection (antioxidation)	↑HO-1, Nqo1, SOD ↑p-Akt ↑p-Nrf2, Nrf2 ↓ROS	Liu Q. et al. (2019)
Protodioscin	PI3K/Akt/Nrf2 pathway	Steroidal saponin	OGD/reperfusion-induced neuronal injury in PC12 cells	Ischemic stroke	Oxidative stress	Neuroprotection (antioxidant, anti-apoptotic effects)	↓ROS, MDA ↑HIF-1α, SOD, GPx HSP70, HO-1, PI3K, pAkt ↑Nuclear Nrf2 ↑miR-124	Shu and Zhang (2019)
Matrine	PI3K/Akt-mediated NF-κB inhibition and Keap1/Nrf2-dependent HO-1 induction	Quinolizidine alkaloid derived from the herb *Radix sophorae* Flavescentis	Subarachnoid hemorrhage in rat	Brain injury	Oxidative stress (secondary effects)	Attenuates neurological deficit, brain edema, and BBB disruption	↓TNF-α, IL-1β↓Bax, caspase-3 ↑Bcl-2 ↑pAkt, pIκB-α↓NF-kB P65 ↑Keap1, Nrf2, and HO-1 ↓MMP-9	Liu et al. (2016)
*Panax notoginseng* saponins	PI3K/Akt/Nrf2 antioxidant signaling pathway	Saponins; *P. notoginseng*	LPS-stimulated cerebral microvascular endothelial cells (bEnd.3)	BBB injury (hemorrhagic stroke)	Oxidative stress	Protection of BBB	↓IL−1β, TNF−α↓ROS ↑Nrf2, HO−1 ↓NF−κB ↑pAkt	Hu et al. (2019)

**TABLE 3:** Anti-aging potentials of phytochemicals that target the PI3K/Akt/Nrf2 signaling pathway.

Modulator	Specific target pathway	Chemical class and natural sources	Experimental model	Disease model	Pathobiology involved	Major research outcomes	Molecular markers	References
Naringenin	PI3K/Akt/Nrf2 Pathway	Polyphenol	D-Galactose-induced brain aging model of male ICR mice	Brain aging	Oxidative stress	Neuroprotection (antioxidant)	↑Nrf2 ↑HO−1, NQO1, SOD, CAT ↑pPI3K, pAkt	Zhang et al. (2017)
Maltol	PI3K/Akt-mediated Nrf2/HO-1 signaling pathway	Maillard reaction product from ginseng	D-Galactose-induced brain aging model of male ICR mice	Brain aging	Oxidative stress	Antiaging (antioxidation)	↑ChAT, ↓AChE ↓ROS, MDA ↑pPI3K, pAkt, ↑pNrf2 ↑HO-1, CAT ↑GSH	Sha et al. (2019)

### Phenolic Compounds

Several phenolics have been reported to exhibit neuroprotective effects against OS in models of AD and other neurodegenerative disorders (Table 1). For example, sulfuretin, a flavonoid glycoside isolated from the stem bark of *Albizia julibrissin* and heartwood of *Rhus verniciflua*, protected SH-SY5Y cells and primary hippocampal neurons from Aβ-induced neurotoxicity (Kwon et al., 2015). The PI3K/Akt and Nrf2/HO-1 signaling pathways have been proposed to contribute to sulfuretin-mediated neuroprotection. Sulfuretin is considered to inhibit cell death by suppressing ROS production and enhancing PI3K/Akt pathway and the nuclear translocation of Nrf2. Resveratrol, a polyphenol found in grapes, and anthocyanins, derived from Korean black beans, protected PC12 cells (Hui et al., 2018) and HT22 cells (Ali et al., 2018), respectively from Aβ-induced toxicity by activating the PI3K/Akt/Nrf2 pathway. In Aβ-induced toxicity, resveratrol inhibited cell death and suppressed OS markers such as MDA and ROS by increasing the phosphorylations of PI3K and Akt, the nuclear translocation of Nrf2, and the protein levels of SOD, HO-1, and GSH (Hui et al., 2018). Anthocyanins attenuate cell death by suppressing the expressions of pro-apoptotic markers (e.g., cleaved caspase-3) and stress markers (MDA, H_2_O_2_, 8-OHdG) and enhancing the phosphorylations of PI3K, Akt, glycogen synthase kinase-3 beta (GSK3β), the nuclear translocation of Nrf2, the expression of HO-1, and GSH levels (Ali et al., 2018). Tea polyphenols (TPs) attenuated OS in H_2_O_2_-stimulated SH-SY5Y cells by activating the Keap1-Nrf2 signaling pathway and the TrkB/CREB/BDNF pathway (Qi et al., 2017). TPs attenuated H_2_O_2_-induced cell death and mitochondrial dysfunction and elevated ROS and H_2_O_2_ levels (Qi et al., 2017). Moreover, TPs enhanced the nuclear translocation of Nrf2 and the TrkB/CREB/BDNF signaling pathway by activating the PI3K/Akt pathway, and thus, transcriptionally regulated the downstream expressions of HO-1, NQO1, SOD, GPx, and CAT in SH-SY5Y cells (Qi et al., 2017). 8-Hydroxydaidzein (8-OHD), an isoflavone of fermented soy, protected against neuroinflammation in LPS-stimulated BV2 microglial cells (Wu et al., 2018) by activating Nrf2-antioxidant and Akt/NF-κB-inflammatory signaling pathways. In BV2 microglial cells, 8-OHD inhibited the LPS-stimulated productions of NO, TNF-α, and IL-6 by suppressing gene expression (Wu et al., 2018). Moreover, 8-OHD quenches ROS and promotes the nuclear translocation of Nrf2, and thus, upregulates the expressions of Phase II enzymes, such as HO-1, NQO1, and GCL (Wu et al., 2018). 8-OHD also suppresses the LPS-stimulated phosphorylations of Akt and NF-κB-p65, and attenuates LPS-induced prostaglandin E_2_ (PGE_2_) production without affecting COX-2 expression (Wu et al., 2018). Rutin, a flavonoid found in buckwheat, protected male albino SD rats from acrylamide or γ-radiation-induced neurotoxicity by activating the PI3K/Akt/GSK-3β/NRF-2 signaling pathway (Thabet and Moustafa, 2018). Here, rutin increased the phosphorylations of PI3K, Akt, and GSK-3β and the nuclear translocation of Nrf2, suppressed MDA levels, GST activity, and the expressions of IL-1b and IL-6, and increased IGF1 and NGF levels (Thabet and Moustafa, 2018).

Three phenolics have been shown to have neuroprotective effects against OS in ischemic stroke and other brain injury models (Table 2). Totarol is a phenolic diterpenoid isolated from the sap of *Podocarpus totara* and protected rat cerebellar granule neurons and cerebral cortical neurons from glutamate and OGD (oxygen-glucose deprivation)-induced injury in a manner involving the Akt/HO-1 pathway (Gao et al., 2015). Totarol increased the nuclear translocation of Nrf2, the expressions of HO-1 and SOD, GSH levels, and the phosphorylations of Akt and GSK-3β (Gao et al., 2015). In CD-1 right middle cerebral artery occlusion (MCAO) mouse model, rosmarinic acid protected against ischemic stroke (Cui et al., 2018) by activating the PI3K/Akt/Nrf2 signaling pathway. In this ischemic model, rosmarinic acid improved ischemic outcomes, attenuated neuronal apoptosis, upregulated the protein and mRNA levels of Bcl-2, HO-1, and Nrf2, downregulated Bax expression, increased SOD activity and Akt phosphorylation, and lowered MDA levels (Cui et al., 2018). Baicalin is a flavone isolated from the radix of *Scutellaria baicalensis*, and in a TBI mouse model protected from oxidative injury by activating the Akt/Nrf2 pathway (Fang et al., 2018). Baicalin reduced Bax and cleaved caspase-3 levels but enhanced Bcl-2 expression and increased the nuclear translocation of Nrf2, the expressions of GPx, SOD, NQO-1, and HO-1, and the phosphorylation of Akt (Fang et al., 2018). Baicalin also improved neurological deficits (Fang et al., 2018).

### Non-phenolic Compounds

Several non-phenolics have been reported to exhibit neuroprotective effects against OS in models of AD and other neurodegenerative disorders (Table 1). For instance, brassicaphenanthrene A isolated from *Brassica rapa* protected HT-22 neuronal cells from glutamate-induced excitotoxicity and upregulated Nrf2-mediated HO-1 expression via PI3K/Akt and JNK regulatory pathways (Lee et al., 2020). Acerogenin A isolated from the stem bark of *Acer nikoense* (a traditional Japanese medicine) protected HT22 cells from glutamate-induced oxidative injury (Lee et al., 2015 activating the PI3K/Akt/Nrf2/HO-1 pathway. Acerogenin A attenuated cell death by suppressing the production of ROS and increasing the nuclear translocation of Nrf2, the expression of HO-1, and the phosphorylation of Akt (Lee et al., 2015). TMC-256C1 isolated from a marine-derived fungus (*Aspergillus* sp. SF6354) protected BV2 microglial cells from LPS-induced inflammatory response and mouse hippocampal HT22 cells from glutamate-induced neurotoxicity (Kim et al., 2016). The PI3K/Akt/Nrf2 pathway has been implicated in the neuroprotective effect of TMC-256C1. TMC-256C1 suppressed the expressions of pro-inflammatory markers (NF-κB (nuclear factor-kappa B), pIκBα (phosphorylated inhibitor of NF-κB alpha), p65, and p50) and inflammatory cytokines (TNF-α (tumor necrosis factor), interleukin (IL)-1β, IL-6, IL-12), and increased the nuclear translocation of Nrf2 and the expression of HO-1 (Kim et al., 2016). TMC-256C1 also suppressed the expression of IPEG2, NO (nitric oxide), COX2 (cyclooxygenase 2), and iNOS (induced nitric oxide synthase) and increased the phosphorylation of Akt (Kim et al., 2016). Polysaccharide extracts (PPE) of *Perilla frutescens* activated PI3K/Akt and Nrf2-mediated HO-1/NQO1 pathways and protected against H_2_O_2_-induced OS in HT22 cells (Byun et al., 2018). PPE attenuated cell injury by suppressing the expressions of Bax, cytochrome C, and caspases-3,-8, and -9, and enhancing the expressions Bcl-2 and Poly [ADP-ribose] polymerase (PARP) (Byun et al., 2018). PPE also increased the phosphorylations of MAPKs (p38, ERK, JNK), PI3K, Akt and p65, decreased NF-κB level and enhanced the nuclear translocation of Nrf2 and the expressions of HO-1 and SOD (Byun et al., 2018). 3,3′-Diindolylmethane is a metabolite of indole-3-carbinol found in Brassicaceae family and was found to attenuate OS in glutamate-induced HT-22 cells by activating the TrkB/Akt pathway (Lee et al., 2019). 3,3′-Diindolylmethane metabolite attenuated the expressions of Bax, cytochrome c, cleaved caspase-3, and AIF (apoptosis-inducing factor), and increased Bcl-2 expression, the phosphorylations of TrkB, Akt, and CREB, and the expressions of HO-1, GCLC, NQO-1, and GPx (Lee et al., 2019). 3,3′-Diindolylmethane also improved cognitive deficits in scopolamine-treated mice (Lee et al., 2019). *N*-Acetyl serotonin (NAS), a melatonin precursor (non-phytochemical) with a stronger antioxidant effect than 3,3′-diindolylmethane, protected neurons from glutamate or H_2_O_2_-induced-OS (Yoo et al., 2017). NAS-mediated neuroprotection was found to involve BDNF/TrkB/CREB signaling and the Akt/Nrf2/antioxidant system (Yoo et al., 2017). NAS inhibited neuronal death by suppressing the expressions of pro-apoptotic markers (e.g., AIF, Bax, calpain, cytochrome c, and cleaved caspase-3, by enhancing pro-survival markers (e.g., Bcl-2), and by increasing the nuclear translocation of Nrf2 and the expressions of antioxidant enzymes (e.g., HO-1 and GCLC) (Yoo et al., 2017). In addition, NAS also ameliorated scopolamine-induced memory impairment and attenuated cell death in CA1 and CA3 brain regions in mice (Yoo et al., 2017).

Several non-phenolics have been reported to have neuroprotective effects against OS in ischemic stroke models (Table 2). For example, in B35 rat neuroblastoma cells, diallyl trisulfide, an organosulfur compound in garlic oil, activated the PI3K/Akt -mediated Nrf2/HO-1 signaling pathway and protected against OGD-induced neuronal injury (Xu et al., 2015). Diallyl trisulfide inhibited the expressions of pro-apoptotic markers (e.g., cleaved caspase-3), OS markers (ROS and MDA), and increased the nuclear translocation of Nrf2, the expression of antioxidant enzymes (e.g., HO-1), and the phosphorylation of Akt (Xu et al., 2015). Oxymatrine, isolated from the Chinese herb *Sophora flavescens* protected P7 SD rats from hypoxic-ischemic brain injury (Ge et al., 2018), activating the Akt/GSK3β/HO-1/Nrf-2 signaling pathway. In experimental rats, oxymatrine increased the nuclear translocation of Nrf2, the phosphorylations of Akt and GSK3β, and HO-1 expression and attenuated neurological deficits (Ge et al., 2018). 6′-*O*-Galloylpaeoniflorin, a galloylated derivative of paeoniflorin isolated from peony root, protected an OGD-induced ischemic PC12 cell model and a CIRI male Wistar rat model against ischemic stroke by activating PI3K/Akt/Nrf2 (Wen et al., 2018). In this study, 6′-*O*-galloylpaeoniflorin attenuated OS and neuroinflammation, improved neurological deficits, inhibited apoptosis by suppressing the expressions of pro-apoptotic markers (e.g., cleaved caspase-3), inhibited inflammatory cytokine (TNF-α, IL-1β), and MDA levels, and increased the nuclear translocation of Nrf2 and SOD expression by increasing Akt phosphorylation (Wen et al., 2018). Ginkgolides A, B and C are diterpene ginkgolides isolated from *Ginkgo biloba* L. and protected PC12 cells from OGD/R-induced ischemic injury and adult male SD rats subjected to MCAO-induced acute cerebral ischemic injury (Zhang et al., 2018) by activating Akt/Nrf2 and Akt/CREB signaling pathways. These ginkgolides inhibit cell death by suppressing the expressions of Bax and cleaved caspase-3, enhancing the phosphorylations of Akt and pCREB, and increasing the nuclear translocation of Nrf2 and HO-1 expression (Zhang et al., 2018). Liu et al. demonstrated ginkgolides protected against ischemic stroke using an OGD-induced SH-SY5Y cell ischemic model and MCAO-induced model of cerebral ischemic injury in male SD rats (Liu Q. et al., 2019). Ginkgolides inhibited ROS production and increased Akt phosphorylation, the nuclear translocation and phosphorylation of Nrf2, and the expressions of HO-1, Nqo1, and SOD (Liu Q. et al., 2019). Protodioscin protected PC12 cells against OGD/R-induced neuronal injury by activating the PI3K/Akt/Nrf2 pathway by increasing the expressions of HIF-1α, SOD, GPx, HSP70, and HO-1, the phosphorylations of PI3K and Akt, the nuclear translocation of Nrf2, and upregulating miR-124, and thus, attenuating OS (Shu and Zhang, 2019).

Non-phenolics also attenuate other brain injuries by targeting the PI3K/Akt/Nrf2 signaling pathway (Table 2). For example, matrine, a quinolizidine alkaloid derived from the herb Radix S*ophorae flavescentis*, protected rats from subarachnoid hemorrhage (Liu et al., 2016) via PI3K/Akt-mediated NF-κB inhibition and Keap1/Nrf2-dependent HO-1 induction. Matrine suppressed the expressions of inflammatory cytokines (TNF-α, IL-1β) and pro-apoptotic markers (Bax and cleaved caspase-3) and enhanced the pro-survival marker Bcl-2 (Liu et al., 2016). Matrine increased nuclear translocation of Nrf2 and HO-1 expression and lowered NF-kB P65 expression by increasing the phosphorylations of Keap1, Akt, and IκB-α (Liu et al., 2016). *Panax notoginseng* saponins were observed to protect against blood–brain barrier (BBB) injury (Hu et al., 2019) by activating the PI3K/Akt/Nrf2 antioxidant signaling pathway. In LPS-stimulated cerebral microvascular endothelial cells of this BBB injury model, saponins attenuated the productions of ROS and inflammatory cytokines (IL−1β, TNF−α), decreased NF−κB levels, and increased the nuclear translocation of Nrf2 HO-1 expression, and the phosphorylation of Akt (Hu et al., 2019).

Phytochemicals have also been reported to confer neuroprotection against OS-induced brain aging by activating the PI3K/Akt/Nrf2 pathway. For instance, naringenin, a polyphenol, protected against OS-induced aging in a D-galactose-induced male ICR mouse model of brain aging by activating the PI3K/Akt/Nrf2 pathway and increasing the nuclear translocation of Nrf2, the expressions of HO−1, NQO1, SOD, and CAT, and the phosphorylations of PI3K and Akt (Zhang et al., 2017). A similar study showed that maltol, a Maillard reaction product of ginseng, protected against brain aging (Sha et al., 2019), activating the PI3K/Akt-mediated Nrf2/HO-1 signaling pathway. In a D-galactose-induced male ICR mouse model of brain aging, maltol inhibited cell death and suppressed the productions of OS markers (ROS and MDA) by enhancing the phosphorylations of PI3K and Akt and increasing the nuclear translocation and phosphorylation of Nrf2 and the expression of antioxidative enzymes, such as HO-1, CAT, and GSH (Sha et al., 2019). Maltol also suppressed AChE and increased ChAT production (Sha et al., 2019).

In addition to the aforementioned reports, Murphy and Park (2017) reviewed the neuroprotective effects of several phytochemicals, such as luteolin (Lin et al., 2010), apigenin (Han et al., 2012; Zhao et al., 2013), 7,8-dihydroxyflavone (Jang et al., 2010), harpagoside (Li et al., 2015), and allicin (Li et al., 2010; Zhu et al., 2015), which all target antioxidant defense and neurotrophic signaling systems (Murphy and Park, 2017). Several natural compounds have been shown to have neuroprotective potential by either activating the antioxidant defense system (Sun et al., 2017), for example, pinocembrin (Jin et al., 2015; Wang et al., 2016), naringenin (Lou et al., 2014), ginsenoside Re (Liu M. et al., 2019), genistein (Wang et al., 2013), orientin (Yu et al., 2015), tiliroside (Velagapudi et al., 2018), or by activating the neurotrophin-mediated cell survival system (Moosavi et al., 2015), for example, curcumin (Zhang et al., 2015), topiramate (Mao et al., 2015), 3β,23,28-trihydroxy-12-oleanene 3β-caffeate (Cheng et al., 2019), and icariside II (Yin et al., 2018).

## Conclusion and Future Directions

Oxidative stress has been implicated in the pathogeneses of degenerative brain disorders, and hence, its targeting offers a means of developing a viable strategy to treat these chronic brain diseases. Cells are equipped with an antioxidant defense system to combat the effects of OS, and Nrf2 is the master regulator of redox homeostasis and does so by activating the antioxidant enzyme system. Accordingly, targeting Nrf2 appears to offer a means of controlling OS. However, attenuating OS alone may not confer sufficient protection against these diseases, in which case, targeting the classical cell survival pathway, that is, the TrkB/PI3K/Akt pathway would be required to restore cellular function, as these signaling pathways upregulate prosurvival factors but suppress their pro-apoptotic counterparts. Pharmacological modulators that can coactivate TrkB signaling-mediated cell survival and Nrf2-ARE antioxidant systems offer promise for the treatment of diseases associated with OS-associated brain degeneration. In this context, several phytochemicals have been reported to protect against neuronal injury by activating TrkB/PI3K/Akt and Nrf2 signaling systems, which suggests they could be utilized to design novel therapeutic agents for NDD, ischemic stroke, TBI, and brain aging.

In addition, being antioxidants, several vitamins such as vitamins E and C and the related compounds have also shown to confer neuroprotection in preclinical studies (Boccardi et al., 2016; Alzoubi et al., 2019). However, the majority of the human trials with these antioxidant vitamins failed to provide compelling evidence of clinical efficacy to improve AD outcomes (NCT00040378; Galasko et al., 2012; Farina et al., 2017; Kryscio et al., 2017). Although the causes are multifactorial, these vitamins only are only suggested to limit OS via directly scavenging reactive free radicals, thus acting as non-specific protective chemical shields (Behl, 1999). No evidence is, so far, suggestive of their capacity to enhance regeneration of damaged neuronal networks that occurred in neurodegenerative disorders and brain injury. On the contrary, phytochemicals (for example, resveratrol, tea polyphenols, and some other compounds mentioned earlier) that were shown to promote the regeneration capacity of neurons along with their protection by dual targeting TrkB/PI3K and Nrf2-ARE signaling (Qi et al., 2017; Hui et al., 2018) may have a better chance of succeeding in the clinical trial with AD subjects.

Although the neuroprotective actions of these phytochemicals are encouraging, their effects have only been reported in preclinical studies. No clinical evidence investigating the neuroprotective potential of phytochemicals that involve TrkB/Nrf2 signaling pathways has, so far, been reported. The attempt to clinical studies may fail to succeed, even if a phytochemical in preclinical investigations responds significantly. The reasons for these outcomes might include the poor bioavailability and the discrepancy between the doses used for the preclinical investigation and those used in clinical trials. Resveratrol, for example, is a potent neuroprotective agent that activates PI3K/Akt/Nrf2 pathway in the cell culture system, but shows poor bioavailability (due to chemical instability, BBB, low absorption, rapid metabolism, and clearance) and thus requires improved drug delivery systems, such as nanoparticle-mediated drug delivery along with the appropriate drug dose regimen. Moreover, the in-depth molecular cross-section of the neuroprotective effects of phytochemicals is essential to identify which cellular defense system between TrkB signaling and Nrf2 pathways is particularly involved in this effect and to elucidate pharmacodynamics.

## Author Contributions

MAH contributed to the design of this review, manuscript writing, table, and figure construction. RD contributed to manuscript writing and figure drawing. AAMS contributed to manuscript writing, revision, and summary table preparation. MNH contributed to manuscript writing and revision. ISM contributed to review planning, supervision, and manuscript revision. All authors contributed to the article and approved the submitted version.

## Conflict of Interest

The authors declare that the research was conducted in the absence of any commercial or financial relationships that could be construed as a potential conflict of interest.
